# Zinc and Copper Metabolism and Risk of Autism: a reply to Sayehmiri et al

**Published:** 2017

**Authors:** Keith FLUEGGE BA

**Affiliations:** Research Scientist, Institute of Health and Environmental Research, Cleveland, USA

**Keywords:** Autism spectrum disorders, Zinc, Copper

## Abstract

**Objective:**

Sayehmiri et al. recently conducted a meta-analysis to explore the relationship between zinc and copper metabolism and autism spectrum disorders (ASD). Recent reports have elucidated a full behavioral profile of mice exposed to prenatal zinc deficiency and documented a phenotype similar to that found in autism spectrum disorders (ASD). These studies suggest that significant alterations in Zn metabolism may be an important nutritional component in the development of ASD.

**Materials & Methods:**

The idea that prenatal zinc deficiency may be to blame is cursorily challenged. Epidemiological studies show that high-income countries with a low estimated prevalence of inadequate zinc intake report the highest prevalence of ASD. Consistent with other reports indicating a link between air pollution and ASD, it has recently been proposed that use of the herbicide, glyphosate, in agriculture may serve as an instrumental variable in predicting later neurodevelopmental impairment via emissions of the agricultural air pollutant, nitrous oxide (N2O).

**Results:**

Work in anesthesiology has demonstrated the neurological effects from subanesthetic doses of N2O, including its inhibition of the alpha 7 nicotinic acetylcholine receptor (α7), a receptor coupled to both central nitric oxide (NO) metabolism and peripheral anti-inflammation.

**Conclusion:**

This correspondence explores how the aforementioned nutritional phenotypes found by Sayehmiri et al. in their systematic review may be a compensatory mechanism to counter the effects (namely, α7 inhibition) of air pollutant exposures occurring during the most critical stages of fetal development.

## Introduction

Sayehmiri et al. (1) recently conducted a meta-analysis to explore the relationship between zinc and copper metabolism and autism spectrum disorders (ASD). The authors found that a significant correlation did exist between levels of Zn and Cu and development of ASD and further implying that the suspected nutritional imbalance may have pathogenic role in ASD development. Consistent with the review conducted by Sayehmiri et al. (1), Grabrucker et al. (2) published a full behavioral profile of mice exposed to prenatal zinc deficiency and presented a phenotype similar to that found in autism spectrum disorders (ASD), reporting increasing anxiety, alterations in nesting and social behaviors, and impaired communication and motor learning.

These studies suggest that significant alterations in Zn metabolism can be considered an important nutritional component in the development of ASD. 

The idea that prenatal zinc deficiency may be to blame is cursorily challenged, however. Epidemiological studies that show that high-income countries with a low estimated prevalence of inadequate zinc intake (3) report the highest prevalence of ASD (4). Furthermore, the idea of maternal nutrient deficiencies conflicts with the elevation of some minerals like Cu in ASD (5). Therefore, it is important to consider that imbalances of trace minerals in ASD may not reflect fetal micronutrient deficiencies resulting from maternal inflammation; rather, these mineral deficiencies may be a complex, compensatory mechanism brought about to counter gestational environmental exposures. 

Consistent with a current line of evidence implicating air pollution - and specifically environmental exposure to the agricultural and combustion pollutant, nitrous oxide (N2O), - as a risk factor in ASD (6,7), it has been previously suggested that N2O-mediated inhibition of the human alpha-7 nicotinic acetylcholine receptor (α7) (8), a ligand-gated ion channel highly permeable to Ca2+ ions which is coupled to neuronal nitric oxide synthase (nNOS) activity (9,10) and implicated in both attention (11) and anti-inflammation (12), would lead to a critical nNOS uncoupling. (7,13). Ex-vivo analysis on cultured brains indicates that neuronally generated NO may stimulate axon pruning yet inhibit neuron regrowth (14), suggesting that the uncoupling of central NO metabolism may contribute to defective pruning found in autism (15). Not only would this central nNOS disruption lead to ADHD symptoms in ASD (16) but profound metabolic changes would also occur, mediated in part by abnormal oxidative stress markers shunted into the periphery in a compensatory exercise to restore central nitric oxide (NO) metabolism (13). Peripheral mechanisms, including altered Zn status in ASD, may follow as a compensatory cascade to re-establish dysregulated central NO metabolism. This argument is supported with the evidence that serum NO levels were found to be significantly higher in ASD than aged and gender-matched controls (17). 

In addition to its role as a blocker of wild-type α7 (18), Cortese-Krott et al. (19) demonstrated using a series of luciferase reporter assays that zinc potently regulates NO output in aortic endothelial cells through its inhibition of the NF-kB-dependent transactivation of the inducible nitric oxide synthase (iNOS) promoter. Cui et al. (20) previously reported that zinc deficiency promoted the induction of IL-1A-mediated iNOS activity in rat intestine, and this effect was rapidly reversed with zinc repletion. Lung injury in adult male rats fed a zinc-deficient diet was characterized by pulmonary edema, lung NO production, higher expression of iNOS and other biomarkers indicative of NF-kappaB activation (21). These anatomic-specific antagonistic relationships are undergirded by the role that Zn plays in NOS dimer stability, which may be mediated by intimate communication between the Zn and tetrahydrobiopterin (BH4) binding sites (22). Since iNOS is the least stable isozyme of the three (23), reduced serum Zn may reflect the role of iNOS in peripheral redox environments (24) such as that imposed from a N2O-mediated impairment in central NO metabolism. 

Whereas Zn possesses anti-inflammatory action in human health (25), Cu exerts a pro-inflammatory effect via substantial upregulation in iNOS activity similar to the inflammatory cascade induced by bacterial LPS (26). Demura et al. (27) clarified that Cu-induced activation of endothelial NOS in human pulmonary arterial endothelial cells was dose-dependent and occurred in the presence of extracellular Ca2+. In isolated rat aortic rings precontracted with phenylephrine, administration of a Cu2+ chelator (diethyldithiocarbamic acid) inhibited smooth muscle relaxation induced by 100 uM sodium nitroprusside (SNP), a NO donor, which was reversed by equimolar concentration of Cu2+ but not Fe2+, suggesting Cu2+ is necessary in SNP-evoked smooth muscle relaxation (28). 

These data suggest that the altered Zn/Cu ratio in ASD reported by Sayehmiri et al. (1) may have functional significance insofar as subsidizing the physiological restoration of impaired central NO metabolism due to chronic, gestational exposure to air pollutants, like N2O. These data argue that Zn deficiencies and Cu excess in ASD may not simply reflect a state of nutritional deficiencies attributable to maternal inflammation from environmental exposures. In fact, the data presented here argue otherwise: that is, gestational exposures to air pollutants may perturb fetal α7 subunit, a receptor that is coupled to central NO metabolism and has been shown to counter peripheral inflammation. Fetal dysregulation of this factor, perhaps among many other known N2O targets, may indirectly potentiate an oxidative stress burden and a fetal inflammatory overload (as characterized by the nutrient profiles herein described) in a vital attempt to overcome dysfunctional central NO metabolism. It is, therefore, critical to thoroughly consider that these nutritional phenotypes may be a purposeful compensatory mechanism (and not just a consequence) to counter air pollutant exposures occurring during the most critical stages of fetal development. Toxicological studies assessing chronic N2O exposure should be undertaken to better understand these potential dynamics herein described. 

Ethical approval: This article does not contain any studies with human participants or animals performed by any of the authors. 

## Author’s Contributions

KF contributed solely to the content of this correspondence. 

## Conflict of Interest:

The author declares no conflict of interest.
